# Creating a Pipeline for Minority Physicians: Medical-Student-Led Programming

**DOI:** 10.7759/cureus.14384

**Published:** 2021-04-09

**Authors:** Vishnu R Muppala, Rohan S Janwadkar, Alicia Rootes, Nirmala Prakash

**Affiliations:** 1 Office of Diversity and Inclusion, Florida Atlantic University Charles E. Schmidt College of Medicine, Boca Raton, USA; 2 Department of Integrated Medical Science, Florida Atlantic University Charles E. Schmidt College of Medicine, Boca Raton, USA

**Keywords:** outreach programs, medical school education, diversity and equity in medicine, community service

## Abstract

Medical School Outreach Programming provides value to medical schools and the community by: (1) fulfilling medical school accreditation requirements, (2) creating pipelines to promote diversity in future healthcare professionals, and (3) providing medical students with opportunities for extracurricular community-level engagement. An Outreach Program initiated at a U.S. community-based medical school provides a medical student-led model with primary goals of improving college candidacy and healthcare career representation for underserved and under-represented minorities in the United States. The Outreach Program also promotes the personal growth and education of medical students.

## Introduction

Medical School Outreach Programming functions to fulfill medical school accreditation requirements, create pipelines for talented, diverse future health professionals, and provide opportunities to engage medical students with opportunities for leadership, extra-curricular learning, and community engagement. Current accreditation standards pertaining to medical education programs leading to the Doctor of Medicine (M.D.) degree contain requirements for community service and/or service learning as designated by the Liaison Committee on Medical Education. The LCME explains these requirements in Functions and Structure of a Medical School [1]. Standard 6.6 details competencies, curricular objectives and curricular design as they relate to service-learning and community service. It states, “The faculty of a medical school ensure that the medical education program provides sufficient opportunities for, encourages, and supports medical student participation in service-learning and/or community service activities [1].” The Outreach Programming at this U.S. Community-Based Medical School fulfills LCME community service/service-learning accreditation standards by hosting science enrichment events for local public schools and giving its medical students the opportunity to lead those events.

The primary aim of the Outreach Programming is to increase racial and ethnic diversity in the next generation of healthcare workers by creating pipeline programming for students in local public schools with interest in pursuing medical career pathways. Community-Based Medical Schools (CBMS) across the United States have a goal of incorporating medical students into the local social and medical communities in which their learning occurs [2]. This type of education model has proven to be a robust learning environment for medical students, which helps them to develop strong communication skills and remarkable clinical reasoning [3], while also engaging with and learning from the local community. By developing interest in science and medicine in the local community, the Outreach Programming allows for a unique way in which the Medical School can directly contribute to the community apart from clinical care. Middle and high school students that participate in the Outreach Program come from economically disadvantaged and diverse racial/ethnic backgrounds. Many students are eligible for free/reduced lunch, reside in homes where English is a second language, and/or have parents who have minimal experience navigating a path to higher education and will be first-generation college students.

The program goals include:

1. Create an enriched 6-12th grade pipeline within the local County Public School System to increase recruitment/retention of minorities and economically disadvantaged students with interest in healthcare education.

2. Improve grade point averages in science to improve college and health professional school candidacy.

3. Strengthen students desire to pursue a career in healthcare as measured by career decidedness surveys.

## Materials and methods

Selection of participants

The Outreach Program serves Title I Schools in the local community; these are schools in which children from low-income families, as determined by the federal poverty line, make up at least 40 percent of total enrollment [4]. In the local community where this Outreach Program was established, most participating schools had populations of nearly 60% of students who were eligible for free and/or reduced lunch and who were low-income [5].

Students were provided information about the Outreach Program by their teachers and by medical students who visited local Title I schools. Students grade 6 through grade 12 submitted an online application for the Outreach Program. The application included demographic information as well as free-response essays to questions pertaining to their interest in Medicine and Healthcare. Teachers also provided recommendations for students. All students who applied to this program must have met the following eligibility criteria: (1) active enrollment in school, (2) a passing score on their respective grade-level state assessments and (3) a passing score on an internal assessment which includes two essays and an interview. Students were then selected based on teacher recommendation, application responses, and demonstrated interest in the program. The Outreach Program has a capacity to accommodate approximately 120 students from each grade level from local Title I schools. In the academic year 2018-2019, there were 1,175 total applications and 823 were selected (70%). These students were then scheduled by their school district bus system to meet at the CBMS campus twice weekly. 

The Outreach Program supports underrepresented and under-served minorities on their path towards Medicine. Students come from economically disadvantaged and diverse racial/ethnic backgrounds. Many students are eligible for free/reduced lunch, reside in homes where English is a second language, have parents who cannot navigate a path to higher education, and will be first-generation college students.

Medical students are given opportunities to volunteer with the Outreach Program beginning in their first year of medical school. Medical students at the CBMS are able to sign up for volunteer shifts based on availability. There are also opportunities for leadership in the Outreach Program that medical students may apply for throughout their medical school career.

Program overview

Students who participate in the Outreach Program engage with biomedical science curriculum which includes college access/prep, mentoring and skill-building activities to enhance creativity, collaborative problem-solving, and self-directed learning. The healthcare-specific activities include medical simulations, dissections, labs, and in-classroom instruction created and led by the CBMS medical students and faculty. This programming enables students to learn biomedical concepts using robust hands-on activities, utilizing the same “high-tech/high-touch” state-of-the-art medical school facilities and curricular innovations used to teach nursing students, medical students, medical residents and first responders. Embedding the Outreach Programming over a longitudinal pathway provides educational, mentorship and enrichment opportunities for local 6-12th grade students, strengthens their confidence in the sciences and promotes retention and success along their educational and health career pathway [6].

Students who participate in the Outreach Programming engage in in-classroom teaching at their respective middle/high school by medical students, in addition to activities on the medical school campus. These activities are aligned to local district curricula and are designed to support different learning styles. The Outreach Program curriculum components all directly mimic teaching techniques and strategies designed by the College of Medicine curriculum. Sample activities include: (1) students deepen their anatomy and disease knowledge through animal dissections and cadaver donor cases led by medical students in the Gross Anatomy Lab, (2) at the medical school simulation center, high-fidelity patient simulators interact with students who learn how to ask relevant medical history questions, obtain vital signs and listen to the heart and lungs for clues to help them determine their patient’s condition, and (3) Outreach Program Saturday Sessions provides longitudinal programming over 10 weeks where selected students learn specialty-specific medicine from healthcare professionals and engage in college preparatory workshops covering essay writing, interviewing, transition to college, and research and presentation skills. Medical students create and coordinate all aspects of Outreach Program Saturday Sessions and can volunteer for the program from their first year of medical school through residency.

Outcomes were measured for the 2019-2020 academic year using pre-assessment and post-assessment surveys. 6th- and 7th-grade students took the Career Decision Scale (CDS) survey, the Quantum Healthcare Career Pipeline Survey, the National Science Foundation (NSF) STEM Semantics Survey, and the STEM Career Interest Survey (STEM-CIS). 8th through 12th graders took these four surveys and the Career Thoughts Inventory (CTI).

Engaging medical students

Medical schools across the United States can achieve effective outreach programming by engaging medical students directly in their formulation and implementation. At the currently discussed Community-Based Medical School, medical students are responsible for planning and implementation of Outreach Program activities. Four student leaders are selected for leadership positions each academic year in which their responsibilities include: (1) Determining activity dates through coordination of medical student, College of Medicine, and local County Public School District calendars, (2) Creation of anatomy lab and clinical skills activities and cases that augment the school district curriculum along with specialty activities, (3) Recruitment and training of medical student volunteers for Gross Anatomy Lab, Clinical Skills Simulation Center, and specialty activities, (4) Oversight of the activities including timekeeping and volunteer management.

Medical student involvement is not only necessary for program functionality, it serves as a mechanism through which medical students are able to interact meaningfully with their medical school curriculum. In both clinical skills and anatomy lab clinical cases, medical students are required to condense and disperse information to make complex medical knowledge accessible to students spanning from sixth to twelfth grade. This material must be integrated with anatomy and physical exam skills during timed sessions which provides additional opportunities for medical students to identify gaps in their knowledge and hone their medical skills, while teaching middle and high school students appropriate medical techniques. Medical students also administer group problem-based learning (PBL) clinical cases which directly mimics the preclinical curriculum at the College of Medicine. In these sessions, medical students function in the role that medical school faculty hold in their own coursework and lead groups of Outreach Program students in PBL cases. Volunteer medical students are also given the opportunity to apply their medical studies to create problem-based learning cases pertaining to clinical skills and anatomy. Engaging with medical school coursework through these various mechanisms facilitates deep understanding and long-term retention of various challenging concepts that form the foundation of preclinical medical education [6-8]. Apart from providing a platform for medical students to interact with real-world presentations of clinical pathologies, this provides a source of unique and current educational content for Outreach Program students.

In addition to the formal curriculum, Outreach Programming provides medical students with opportunities to be informal mentors as part of the “hidden curriculum” built into the program. Program participants meeting with medical students many times during their secondary education and form mentoring relationships which extend beyond the program duration and into post-secondary education. An additional mentoring mechanism built into the Outreach Program permits pre-health undergraduate students to assist medical students in a new, informal pipeline from the local chapter of the American Medical Student Association (AMSA). Twenty-one AMSA students selected from the general membership of the local Chapter operated in supervised volunteer roles where they were trained in medical aspects related to program activities and transitioned to leading activities under supervision.

## Results

During the 2018-2019 academic year, 54 different medical students volunteered a combined total of 470 hours and engaged with 823 educationally disadvantaged/under-represented minority middle and high school students through Outreach Programming (Tables 1, 2). The program is both grant-funded and revenue-generating with funds covering all programmatic and personnel costs. Since 2014, medical students have led more than 4,000 students from 20 schools through simulations, dissections, donor cases, and PBL cases and have led up to 32 independent Outreach Program activities from September through February each year. AMSA pre-medical undergraduates volunteered 270 hours with Outreach Programming this past academic year.

**Table 1:** Program participant demographics AY18- 19. AY: academic year.

Sex	n (%)
Female	533 (64.8)
Male	169 (20.5)
Not reported	131 (14.7)
Race	n (%)
White	77 (9.4)
Black	292 (35.5)
Hispanic	233 (28.3)
Asian or Pacific islander	57 (6.9)
Native American	2 (0.2)
Multi-racial	13 (1.4)
Other	3 (0.4)
Not reported	146 (17.8)

**Table 2:** Program volunteer demographics.

Medical students	
Sex	n (%)
Female	33 (61.1)
Male	21 (38.9)
Race	n (%)
White	27 (50.0)
Black	4 (7.5)
Hispanic	5 (10.0)
Asian or Pacific islander	18 (32.5)
Pre-medical students	
Sex	n (%)
Female	14 (66.7)
Male	7 (33.3)
Race	n (%)
White	4 (20.8)
Black	7 (33.3)
Hispanic	6 (25.0)
Asian or Pacific islander	4 (16.7)
Medical residents	
Sex	n (%)
Female	1 (50.0)
Male	1 (50.0)
Race	n (%)
White	1 (50.0)
Black	1 (50.0)
Medical faculty	
Sex	n (%)
Female	7 (70)
Male	3 (30)
Race	n (%)
White	6 (60.0)
Black	3 (30.0)
Asian or Pacific islander	1 (10.0)

Current Outreach Program outcomes include significant gains in medical career certainty by 8th grade, suggesting after three years of HCOP activities, students have gained confidence in their career choice and their capability in this rigorous course of study. By 12th grade, we see a significant difference in career certainty (increase) and career indecision (decrease). Graduating seniors showed an increased connection to the field of healthcare, feelings of confidence in their learning, and increased STEM career decision-making. The intervention showed effectiveness in retaining students in STEM in 9th-12th grade and provides a learning pathway to engender STEM-persistence.

Under the Outreach Program umbrella, medical students also volunteer with Outreach Program Saturday Sessions. Forty-five individual medical students and residents have volunteered in this capacity and 122 out of 128 juniors and seniors matriculating from community high schools have completed the program (95% completion rate).

## Discussion

Despite the changing demographics of the United States, medical school graduates by race and ethnicity have remained consistent over time. According to the most recent report by the Association of American Medical Colleges, underrepresented minorities in medicine have lower medical school acceptance rates than peer applicants, and the demographics of medical school matriculants and graduates demonstrate a substantial gap between the proportion of URM students in medical education and the general population makeup of the United States [9]. Acceptance rates for medical school admission for Black or African American applicants and Hispanic and Latino applicants were 34% and 42% respectively. For White and Asian applicants, the medical school acceptance rates were 44% and 42%. Whites (58.8%) and Asians (19.8%) continue to comprise the largest proportion of medical student graduates while Black or African Americans (5.7%) and Hispanic or Latinos (4.6%) have much lower representation in medical school graduate classes. Additionally, even upon acceptance to medical school, URM medical students have lower retention rates when compared to White medical student peers. This pattern of disproportionally low representation extends beyond medical students as well because similar trends extend to medical school faculty demographics [10].

Racial and ethnic disparities extend beyond medical school acceptance rates and faculty demographics, with minority groups in the United States facing an inequitable risk of lacking access to care and suffering worse health outcomes from preventable and treatable conditions. One of the foremost strategies in reducing disparities is creating racial/ethnic diversity in the health-care workforce in order to improve the quality of care received by minority groups. Diversity in the health-care workforce has been well correlated with the delivery of quality care to minority populations [11].

Dr. Paul B. Rothman, Dean of the Medical Faculty and CEO of Johns Hopkins Medicine, states:

“This is not just about fairness-diversity in medicine has measurable benefits.

Studies show that students trained at diverse schools are more comfortable treating patients from a wide range of ethnic backgrounds. When the physician is the same race as the patient, patients report higher levels of trust and satisfaction. The visits even last longer-by 2.2 minutes, on average. When patients enter our hospitals, they want to see staff members and physicians who resemble them.

All of this matters if we are going to start chipping away at the troubling health disparities we see in this region [12]."

## Conclusions

Health profession pipeline programming targeting middle and high school students is critical to improving minority representation in healthcare, promoting access to higher education, and addressing community-level health disparities. Outreach Programming at Community-Based Medical Schools prepares middle and high school students in the community to successfully pursue careers in medicine through an innovative biomedical science curriculum developed and delivered by medical students, faculty and staff. The programs’ goals are to remove barriers to higher education for the diverse students of the local community and to inspire them to pursue careers in medicine or biomedical research. Pipeline programs will possibly serve to improve URM medical student disparities for the next generation of medical professionals. There are various mechanisms by which the program does this including providing education for college preparedness and pre-health professional track advising, teaching medical knowledge and skills, and creating opportunities for informal mentorship between medical and high school students. Through these mechanisms, we can expect that students will enter college and medical school with increased ability and confidence promoting success, retention and achievement.
